# Effects of tumor necrosis factor-α inhibition on kidney fibrosis and inflammation in a mouse model of aristolochic acid nephropathy

**DOI:** 10.1038/s41598-021-02864-1

**Published:** 2021-12-08

**Authors:** Shinya Taguchi, Kengo Azushima, Takahiro Yamaji, Shingo Urate, Toru Suzuki, Eriko Abe, Shohei Tanaka, Shunichiro Tsukamoto, Daisuke Kamimura, Sho Kinguchi, Akio Yamashita, Hiromichi Wakui, Kouichi Tamura

**Affiliations:** 1 Department of Medical Science and Cardiorenal Medicine, Yokohama City University Graduate School of Medicine, 3-9 Fukuura, Kanazawa-ku, Yokohama, Japan; 2 Cardiovascular and Metabolic Disorders Program, Duke-NUS Medical School, Singapore, Singapore; 3 Department of Investigative Medicine, Graduate School of Medicine, University of the Ryukyus, Okinawa, Japan

**Keywords:** Chronic kidney disease, Pharmacology

## Abstract

Tumor necrosis factor (TNF)-α is a potent mediator of inflammation and is involved in the pathophysiology of chronic kidney disease (CKD). However, the effects of TNF-α inhibition on the progression of kidney fibrosis have not been fully elucidated. We examined the effects of TNF-α inhibition by etanercept (ETN) on kidney inflammation and fibrosis in mice with aristolochic acid (AA) nephropathy as a model of kidney fibrosis. C57BL/6 J mice were administered AA for 4 weeks, followed by a 4-week remodeling period. The mice exhibited kidney fibrosis, functional decline, and albuminuria concomitant with increases in renal mRNA expression of inflammation- and fibrosis-related genes. The 8-week ETN treatment partially but significantly attenuated kidney fibrosis and ameliorated albuminuria without affecting kidney function. These findings were accompanied by significant suppression of interleukin (IL)-1β, IL-6, and collagen types I and III mRNA expression. Moreover, ETN tended to reduce the AA-induced increase in interstitial TUNEL-positive cells with a significant reduction in Bax mRNA expression. Renal phosphorylated p38 MAPK was significantly upregulated by AA but was normalized by ETN. These findings indicate a substantial role for the TNF-α pathway in the pathogenesis of kidney fibrosis and suggest that TNF-α inhibition could become an adjunct therapeutic strategy for CKD with fibrosis.

## Introduction

Chronic kidney disease (CKD) is increasing worldwide^1^. Because the risk for cardiovascular events increases as CKD progresses^2^, preventing progression is essential for improving the disease prognosis. Kidney fibrosis, the final common pathway in CKD, is characterized by the replacement of functional renal tissue with extracellular matrix^3^, and provides a greater contribution to the risk for end-stage renal disease than glomerulosclerosis, which is another typical pathology of advanced CKD^4^. Kidney fibrosis is initiated by various tubular cell injuries, including ischemia, proteinuria, and exposure to toxic substances^5–8^. Injured tubular cells show cell cycle arrest, de-differentiation, senescence, and increased production of proinflammatory and profibrotic factors, resulting in activation and proliferation of myofibroblast and matrix secretion^5,8^. Several factors that contribute to the development of kidney fibrosis have been identified, however, they have not yet been translated into therapeutic agents.

The transforming growth factor (TGF)-β pathway is a master regulator of kidney fibrosis^9,10^, but inflammation also plays an important role in its progression^3,11,12^. Tumor necrosis factor (TNF)-α is a potent mediator of the inflammatory response produced by various cells, including macrophages, mesangial cells, and tubular epithelial cells^13^. The main pathways activated by TNF-α are centered on caspase, nuclear factor-κB (NF-κB), and mitogen-activated protein kinase (MAPK). The NF-κB and MAPK signaling pathways induce secondary responses by increasing the expression levels of several proinflammatory cytokines, further activating TNF-α^14^. High serum and renal levels of TNF-α have been reported in human CKD and experimental kidney disease, including unilateral ureteral obstruction (UUO), ischemia–reperfusion injury (IRI), and cisplatin-induced nephropathy models^13,15–17^. Furthermore, high serum levels of TNF-α are positively correlated with the severity of kidney injury^15^. Pharmacological and genetic inhibition of TNF-α ameliorates kidney injury in diabetic nephropathy and IRI models in which inflammation plays a critical role in the pathogenesis^18–20^. On the other hand, to elucidate the pathogenesis of the TNF-α pathway in the development of kidney fibrosis, several previous studies have employed the UUO model which is the most widely used mouse model of kidney fibrosis. In these studies, TNF-α inhibition using the pegylated form of soluble TNF receptor type 1 or genetic ablation of TNF receptors successfully had an anti-fibrotic effect against kidney fibrosis induced by the UUO model, indicating that the TNF-α pathway is a promising therapeutic target for kidney fibrosis^21–25^. However, the UUO model has potential limitations, such as the inability to assess changes in kidney function and the rapid progression to kidney fibrosis caused by hydronephrosis, which differs from the process of kidney fibrosis typically observed in human CKD^26^.

In this study, we employed aristolochic acid (AA)-induced nephropathy (AAN) as another model of kidney fibrosis to corroborate the findings reported for the UUO model. AAN is caused by direct cytotoxicity to renal tubular cells and is characterized by progressive tubular atrophy accompanied by chronic interstitial inflammation and fibrosis^27–33^. To investigate whether TNF-α inhibition prevents the development of kidney fibrosis, and to elucidate the underlying molecular mechanisms, we evaluated the effects of etanercept (ETN), which is a fusion protein competitively acting as a "TNF-α decoy receptor" to inhibit the binding of TNF-α to its cell surface receptor^34^, on kidney functional decline, inflammation, and fibrosis in a murine model of AAN.

## Results

### Etanercept does not affect body weight, tissue weight, systolic blood pressure, or heart rate

**Figure 1:**
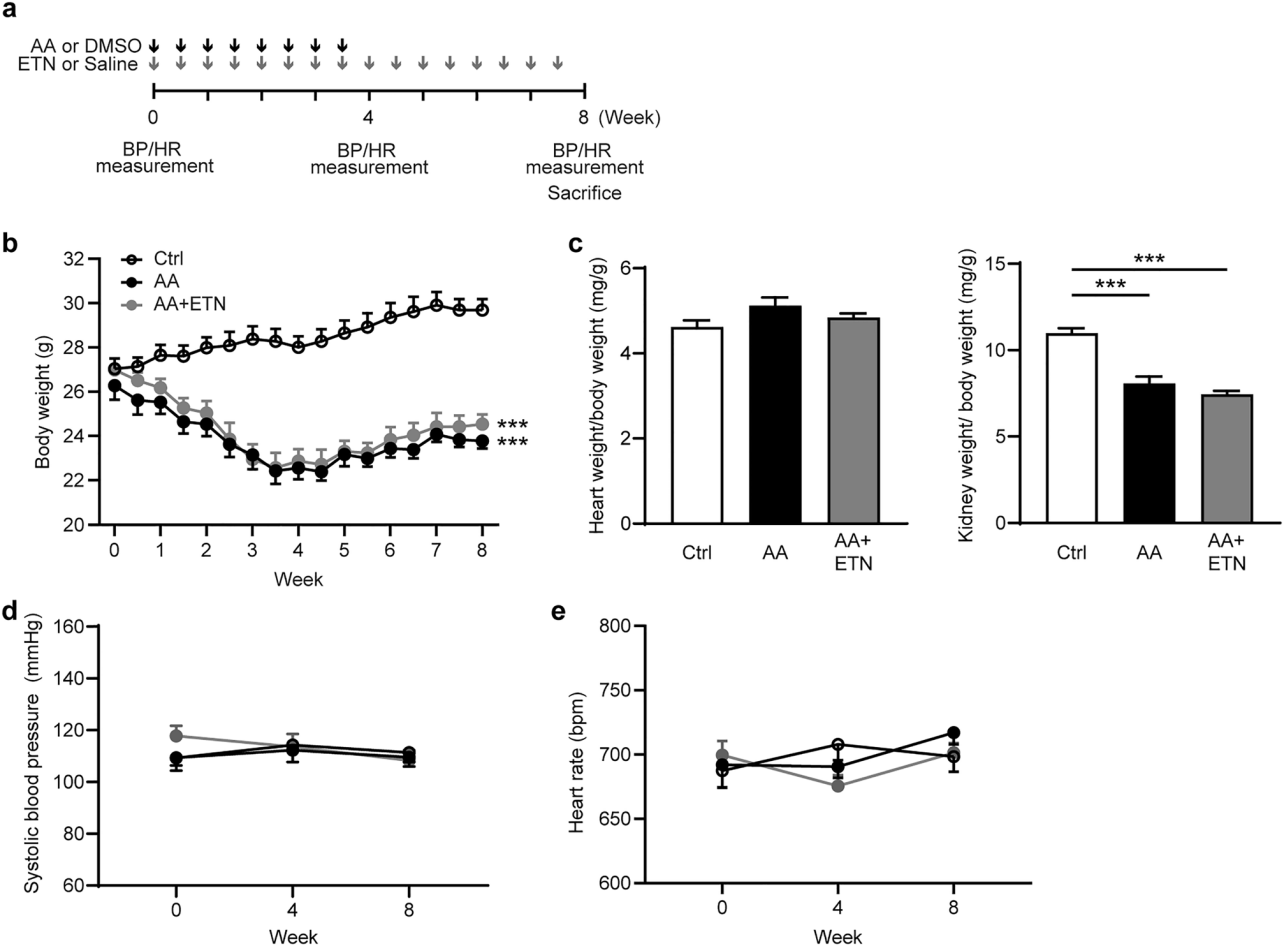
Effect of etanercept on body weight, tissue weight, systolic blood pressure and heart rate. (**a**) Experimental protocol. Control group received DMSO i.p. injection twice per week for 4 weeks and normal saline s.c. injection twice per week for 8 weeks. AA group received AA (3 mg/kg) i.p. injection twice per week for 4 weeks and normal saline s.c. injection twice per week for 8 weeks. AA + ETN group received AA (3 mg/kg) i.p. injection twice per week for 4 weeks and ETN (5 mg/kg) s.c. injection twice per week for 8 weeks. Body weight was measured twice per week for 8 weeks. Blood pressure and heart rate were measured at 0, 4, and 8 weeks after the start of the experiment. (**b**) Changes in body weight. (**c**) Heart and kidney weight to body weight ratio. (**d**) Changes in systolic blood pressure. (**e**) Changes in heart rate. Values are means ± SEM, *n* = 8. *** *P* < 0.001. AA, aristolochic acid; DMSO, dimethyl sulfoxide; ETN, etanercept; BP, blood pressure; HR, heart rate; Ctrl, control.

The changes in body weight (BW), tissue weight, systolic blood pressure (BP), and heart rate (HR) are shown in Fig. 1b-d. At the start of the experimental period, there were no significant differences in BW, systolic BP, or HR among control, AA and AA + ETN groups. BW in the AA and AA + ETN groups decreased during the 4 weeks of AA administration and increased after AA discontinuation (Fig. 1b). At the end of the experimental period, BW was significantly lower in the AA and AA + ETN groups than in the control group (*P* < 0.001 and *P* < 0.001, respectively). There were no significant differences in heart to BW ratio among the three groups (Fig. 1c), but the kidney to BW ratio was significantly decreased in the AA and AA + ETN groups compared to the control group (both *P* < 0.001). There were no significant differences in BP and HR among the three groups (Fig. 1d-e).

### Etanercept does not affect plasma creatinine, blood urea nitrogen, or creatinine clearance but reduces albuminuria

**Figure 2:**
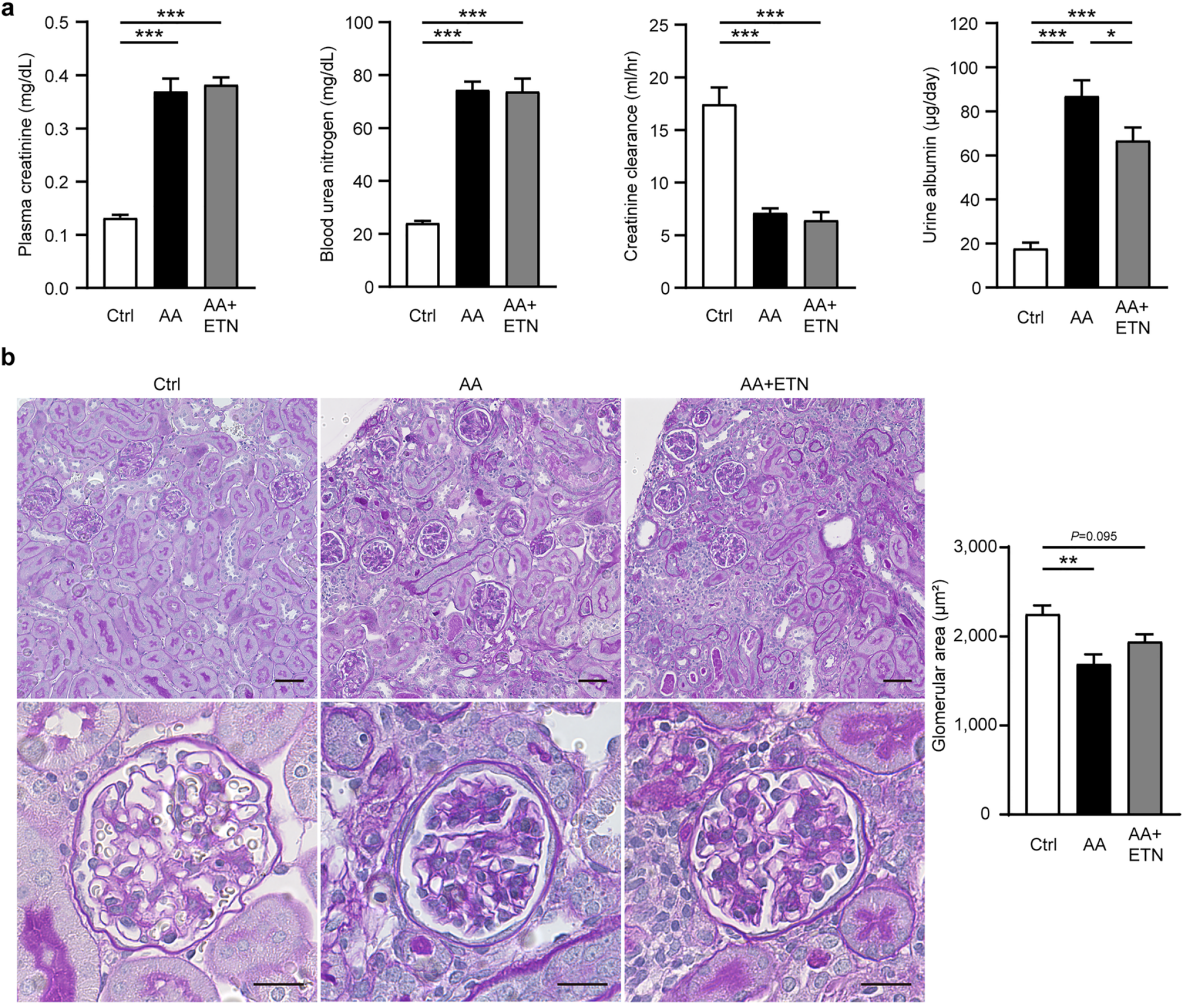
Effect of etanercept on renal function and pathological changes. (**a**) Kidney function as determined by plasma creatinine, blood urea nitrogen, creatinine clearance, and urine albumin (*n* = 7–8). (**b**) Representative images of kidneys stained with PAS (upper panel, original magnification, × 200, bar, 50 μm; lower panel, original magnification, × 400, bar, 20 μm), and quantitative analysis of glomerular area (*n* = 6–8). Values are means ± SEM. * *P* < 0.05, ** *P* < 0.01, *** *P* < 0.001. Ctrl, control; AA, aristolochic acid; ETN, etanercept; PAS, periodic acid Schiff.

Parameters of kidney function are shown in Fig. 2a. Plasma creatinine (Cr) and blood urea nitrogen (BUN) levels were significantly increased in the AA and AA + ETN groups compared to the control group (Cr, *P* < 0.001 and *P* < 0.001; BUN, *P* < 0.001 and *P* < 0.001, respectively). In addition, creatinine clearance levels were significantly decreased in the AA and AA + ETN groups compared to the control group (both *P* < 0.001). There were no differences between the AA and AA + ETN groups in these parameters. Urinary albumin excretion in the AA and AA + ETN groups was significantly increased compared to the control group (both *P* < 0.001); however, urinary albumin excretion in the AA + ETN group was significantly decreased compared to the AA group (*P* = 0.047). In the pathological evaluation, the glomerular area in the AA group was significantly decreased, and that in the AA + ETN group tended to be decreased compared to the control group (*P* = 0.001 and *P* = 0.095, respectively) (Fig. 2b).

### Etanercept partially reduces kidney fibrosis without affecting TGF-β expression

**Figure 3:**
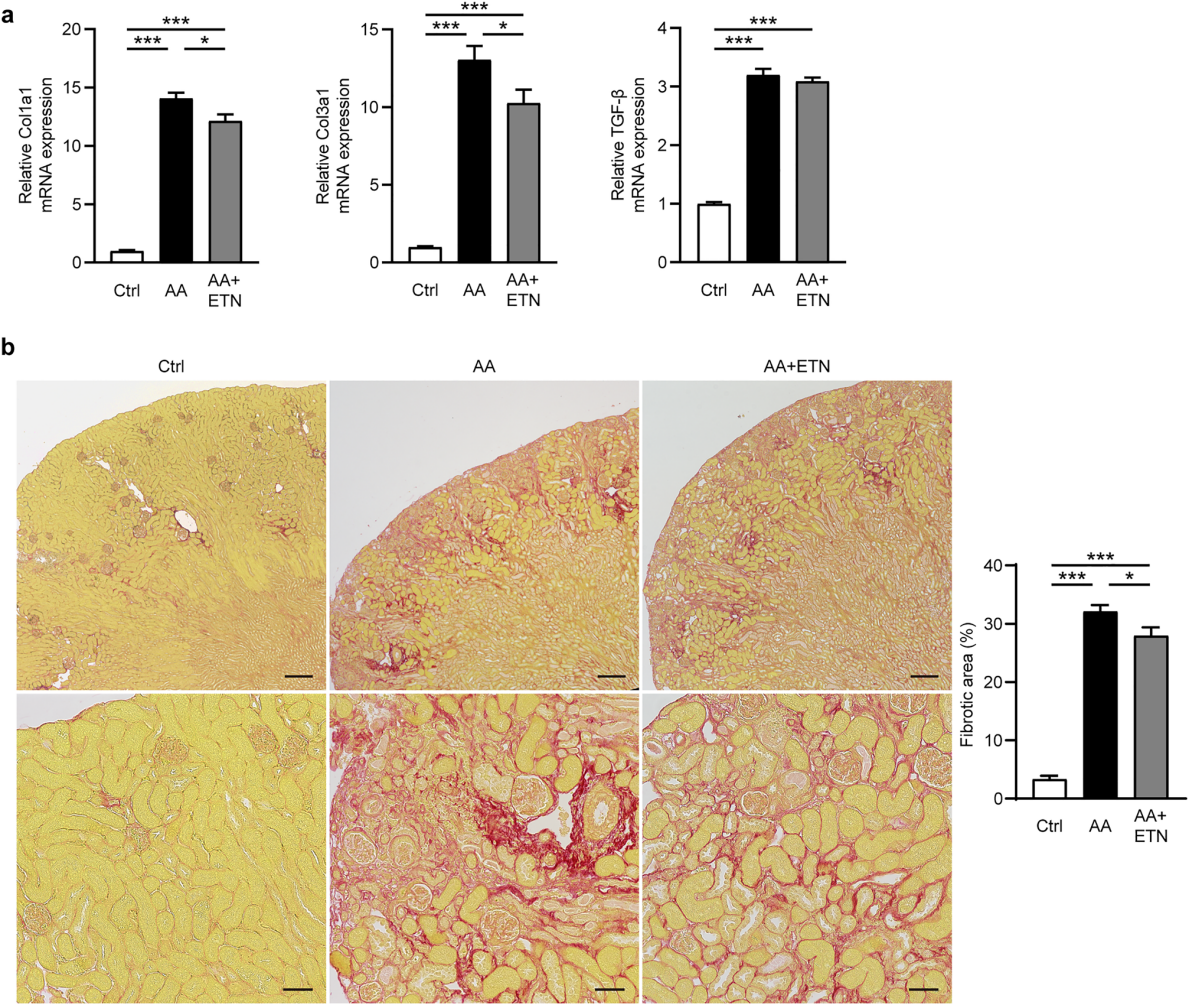
Effect of etanercept on kidney fibrosis. (**a**) Relative renal mRNA expression of Col1a1, Col3a1, and TGF-β (*n* = 7–8). (**b**) Representative images of kidneys stained with picrosirius red (upper panel, original magnification, × 40, bar, 200 μm; lower panel, original magnification, × 200, bar, 50 μm), and quantitative analysis of kidney fibrotic area (*n* = 7–8). Values are means ± SEM. * *P* < 0.05, *** *P* < 0.001. Ctrl, control; AA, aristolochic acid; ETN, etanercept; Col, collagen; TGF, transforming growth factor.

Renal mRNA expression levels of type I and III collagen, major components of the extracellular matrix in the kidneys, were significantly increased in the AA and AA + ETN groups compared to the control group (both *P* < 0.001) (Fig. 3a). However, the increased expression of type I and III collagen was partially but significantly attenuated by ETN (*P* = 0.011 and *P* = 0.012, respectively). Renal mRNA expression of TGF-β1, a pivotal profibrotic cytokine^9^, was significantly and similarly increased in the AA and AA + ETN groups compared to the control group (*P* < 0.001 and *P* < 0.001, respectively). In the pathological evaluation, AA administration caused severe kidney fibrosis, indicated by an increased area of picrosirius red (PSR) staining; however, ETN partially but significantly reduced kidney fibrosis (Fig. 3b). The quantitative assessment showed a significant decrease in the area of kidney fibrosis in the AA + ETN group compared to the AA group (*P* = 0.030). In a preliminary experiment in which we administered ETN at a lower dose (1 mg/kg, twice per week), the expression of type I and III collagen and pathological kidney fibrosis were comparable between the AA and AA + ETN groups (see Supplementary Fig. S1 online).

### Etanercept reduces proinflammatory cytokine expression and phosphorylated p38-MAPK

**Figure 4:**
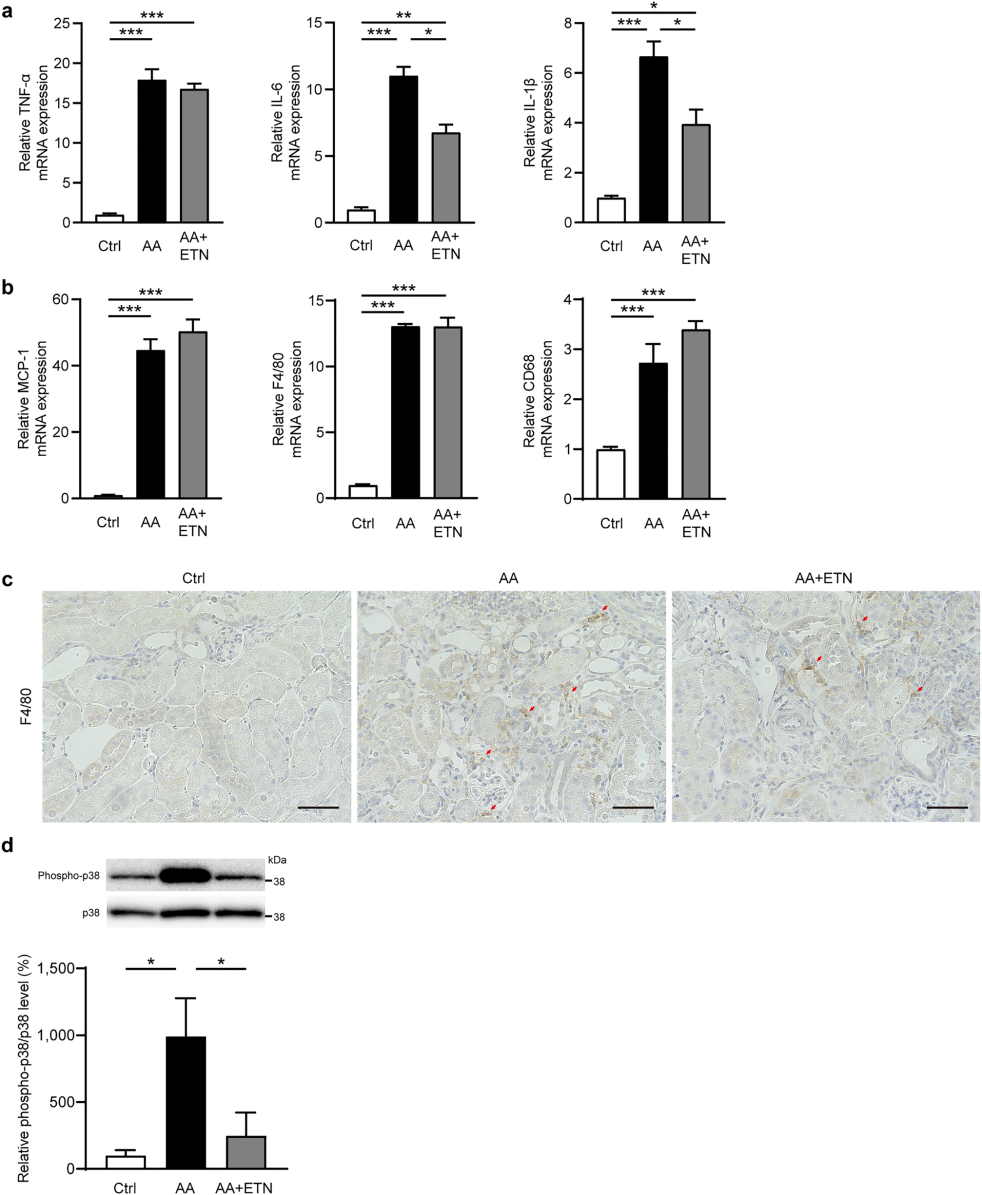
Effect of etanercept on renal inflammation and phosphorylation of p38 MAPK. (**a**) Relative renal mRNA expression of TNF-α, IL-6, and IL-1β (*n* = 7–8). (**b**) Relative gene expressions of MCP-1, F4/80, and CD68 (*n* = 7–8). (**c**) Representative images of kidneys stained with F4/80 (original magnification, × 400, bar, 50 μm). (**d**) Representative western blots and quantitative analysis of phosphorylated and total p38 MAPK in the kidney (*n* = 8). Values are means ± SEM. * *P* < 0.05, ** *P* < 0.01, *** *P* < 0.001. Ctrl, control; AA, aristolochic acid; ETN, etanercept.

To determine how etanercept suppressed kidney fibrosis induced by AA, we examined the mRNA expression of genes involved in inflammation (Fig. 4a-b). AA administration elicited remarkable increases in renal TNF-α, interleukin (IL)-6, and IL-1β expression (all *P* < 0.001). Although there were no significant differences in renal TNF-α expression between the AA and AA + ETN groups, renal IL-6 and IL-1β expression was significantly decreased in the AA + ETN group compared to the AA group (*P* = 0.026 and *P* = 0.016, respectively) (Fig. 4a). Regarding macrophage infiltration, renal MCP-1, F4/80, and CD68 expression were markedly and similarly increased in the AA and AA + ETN groups (both *P* < 0.001). Immunohistostaining of F4/80 revealed obvious renal interstitial macrophage infiltration in the AA and AA + ETN groups (Fig. 4c). Because MAPK pathways are downstream of the TNF-α pathway and are involved in the pathogenesis of kidney fibrosis ^14,35^, we examined p38 MAPK protein levels in the kidneys. The phosphorylated p38 MAPK to p38 MAPK ratio was significantly elevated in the AA group compared to the control group (*P* = 0.011) but was normalized by ETN (*P* = 0.034) (Fig. 4d).

### Etanercept tends to suppress renal interstitial cell apoptosis, and reduces Bax expression

**Figure 5:**
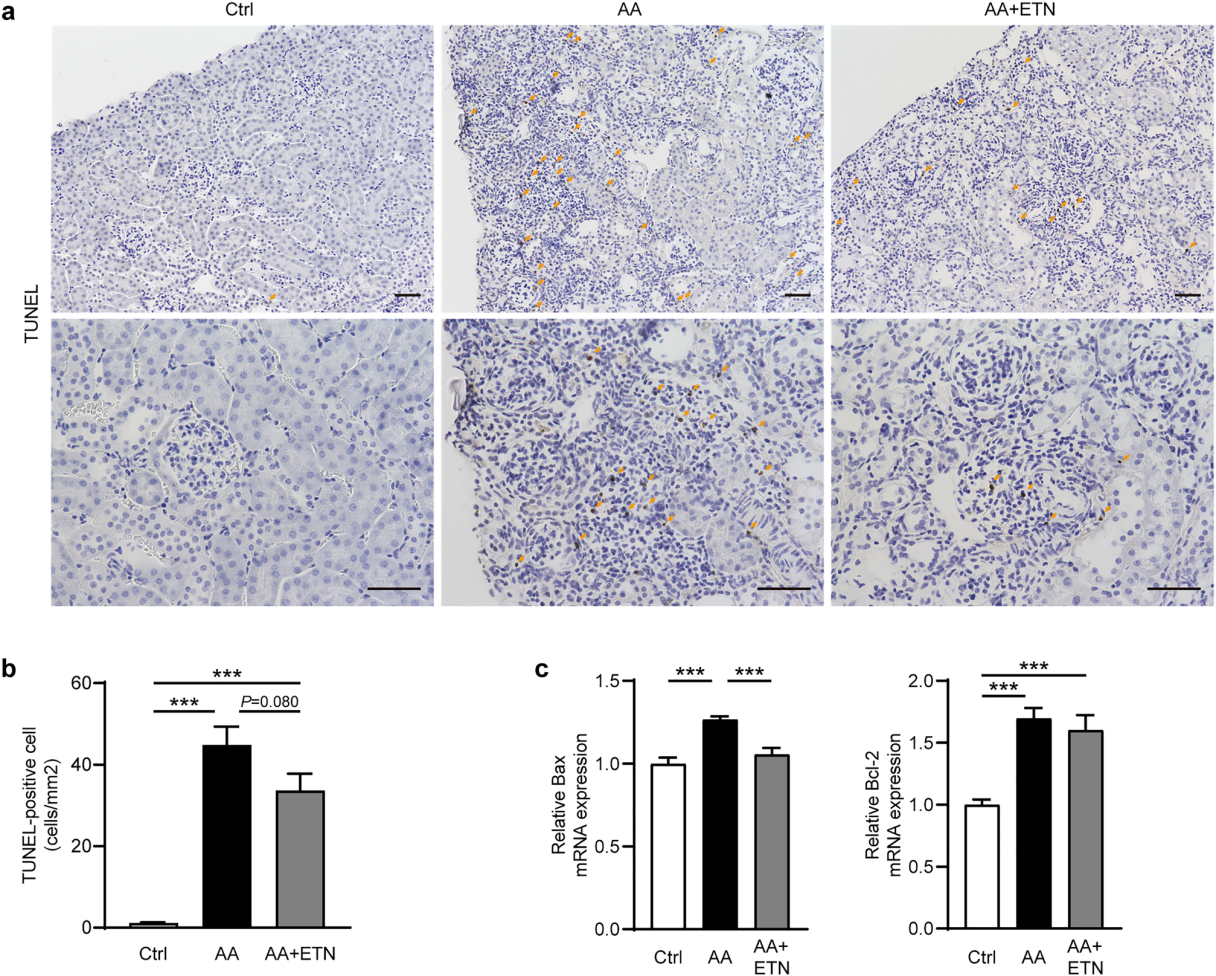
Effect of etanercept on apoptosis. (**a**) Representative images of TUNEL-stained kidneys (upper panel, original magnification, × 200, bar, 50 μm; lower panel, original magnification, × 400, bar, 50 μm). (**b**) Analysis of the number of TUNEL-positive cells. (*n* = 7–8). (**c**) Relative renal mRNA expression of Bax and Bcl-2 (*n* = 7–8). Values are means ± SEM. *** *P* < 0.001. Ctrl, control; AA, aristolochic acid; ETN, etanercept; TUNEL, TdT-mediated dUTP nick-end labeling.

To investigate the mechanism of the anti-fibrotic effect of TNF-α inhibition, we performed a TdT-mediated dUTP nick-end labeling (TUNEL) assay and evaluated apoptosis-related factors. Although the number of TUNEL-positive cells was significantly increased in the AA and AA + ETN groups compared to the control group (both *P* < 0.001) (Fig. 5a-b), ETN tended to reduce the number of TUNEL-positive cells but this trend did not reach statistical significance (*P* = 0.080). Renal expression of Bax, a pro-apoptotic factor, was significantly increased in the AA group compared to the control group (*P* < 0.001) but was normalized by ETN (*P* < 0.001) (Fig. 5c). The anti-apoptotic factor Bcl-2, activated by AA, was not affected by ETN.

## Discussion

We demonstrated that TNF-α inhibition by ETN attenuated kidney inflammation, fibrosis, and albuminuria in a mouse model of AAN. TNF-α inhibition suppressed the AA-induced increase in renal expression of inflammation- and fibrosis-related genes, including IL-1β, IL-6, and type I and III collagen. Moreover, TNF-α inhibition tended to reduce the AA-induced increase in renal interstitial TUNEL-positive cells and significantly reduced the AA-induced increase in renal Bax mRNA expression, a pro-apoptotic factor. These findings imply that the TNF-α pathway plays a substantial role in the development of kidney fibrosis, and that TNF-α inhibition may exert its anti-fibrotic effect by suppressing kidney inflammation and renal interstitial cell apoptosis.

p38 MAPK is downstream of the TNF-α signaling pathway, and its activation promotes apoptosis and the production of proinflammatory cytokines, including TNF-α, IL-1β, and IL-6^13,14,36^. In this study, the AA-induced increase in renal phosphorylated p38 MAPK was normalized by ETN, suggesting that ETN attenuated the upregulated renal TNF-α pathway in the AAN model. This finding is consistent with the result that the AA-induced increase in renal expression of proinflammatory cytokines was significantly attenuated by ETN. Nonetheless, the renoprotective effect of ETN was limited to partial improvement of fibrosis and albuminuria. These results may be associated with the TGF-β pathway, macrophage infiltration, or the method of inhibiting the TNF-α pathway.

In this study, the AA-induced increase in renal TGF-β expression was not affected by ETN. The TGF-β pathway is a master regulator of fibrosis that promotes the epithelial-mesenchymal transition and production of extracellular matrix protein via the Smad-signaling pathway^9,10^. Therefore, the lack of a preventive effect of ETN on upregulation of the renal TGF-β pathway may explain its limited anti-fibrotic effect in the AAN model. Notably, previous studies on the roles of the TNF-α and p38 MAPK pathways in the development of kidney injury also demonstrated that inhibiting these pathways does not attenuate renal TGF-β expression^22,35,37,38^, indicating that the TGF-β pathway is less influenced by inhibiting the TNF-α pathway. No study has evaluated the effects of inhibiting both TNF-α and TGF-β signaling on kidney fibrosis, and future studies addressing this issue might make the anti-fibrotic effect of TNF-α inhibition shown in this study more prominent.

Macrophages play a pathogenic role in the development of kidney inflammation and fibrosis^11,39^. Various strategies, including depletion or repletion of macrophages and blockade of chemokines or their receptors, have demonstrated a pivotal role for macrophages in kidney diseases^40–46^. However, in the present study, ETN did not affect renal interstitial macrophage infiltration induced by AA, and the renal gene expression levels related to macrophage infiltration, including MCP-1, F4/80, and CD68, were not affected by TNF-α inhibition. Previous studies on the effects of TNF-α inhibition in kidney injury models have also reported a lack of improvement of macrophage infiltration. For example, Meldrum et al*.* showed that administering PEG-TNFR1 has no effect on macrophage infiltration in a rat model of UUO^25^. Similarly, Misaki et al*.* reported that administering ETN reduces interstitial mononuclear cell infiltration 3 days after, but not 7 and 14 days after UUO^47^. The reason that ETN did not affect renal interstitial macrophage infiltration in the AAN model is unclear, but insufficient suppression of macrophage infiltration may explain the limited anti-fibrotic effects of ETN.

Genetic ablation of TNF-α receptors has been employed in several studies as another way to inhibit the TNF-α pathway^21,22^. In these studies, genetic ablation of TNF-α receptors had a certain anti-fibrotic effect (20–30% reduction) against UUO-induced kidney fibrosis, which seemed to be more prominent than the results of our study. Therefore, the limited renoprotective effect in this study might be associated with an insufficient ability of ETN to inhibit the TNF-α pathway.

In this study, ETN partially but significantly attenuated the AA-induced increase in albuminuria, whereas it did not affect structural changes in the glomerulus. Because AA causes proximal tubular epithelial cell death without affecting the glomerulus, the AAN model exhibits mild albuminuria, which is considered tubular proteinuria^29^. Given that ETN had its renoprotective effect mainly in the renal interstitium, the partial reduction in AA-induced albuminuria may reflect the degree of improvement in renal interstitial damage by TNF-α inhibition.

This study has limitations. First, an ETN group in which mice received an i.p. injection of DMSO and an s.c. injection of ETN was lacking. Therefore, it is unknown whether TNF-α inhibition has any effect on normal kidneys. Second, because ETN did not improve the AA-induced reduction in creatinine clearance, it is unclear whether TNF-α inhibition has a renoprotective effect on kidney functional decline in the AAN model. This absence of an effect may be attributed to the experimental design such as the experimental period, doses of ETN, or the kidney injury model. Studies using longer and/or different doses of ETN and other kidney injury models, such as adenine-infusion and 5/6 nephrectomy, are needed to address this issue. Third, although the present study demonstrates the anti-fibrotic effect of ETN by suppressing inflammation and apoptosis and inhibiting p38 MAPK signaling, it is unclear which factor contributed more prominently.

In conclusion, our findings show that TNF-α inhibition by ETN partially attenuates the development of kidney fibrosis and albuminuria by suppressing kidney inflammation and interstitial cell apoptosis in a mouse model of AAN. Our results support evidence of an anti-fibrotic effect of TNF-α inhibition on kidney fibrosis that has mainly been established by the UUO model. Collectively, these findings indicate a substantial role for the TNF-α pathway in the development of kidney fibrosis and imply that TNF-α inhibition could become an adjunct therapeutic strategy for CKD with fibrosis.

## Methods

### Animals

This study was performed following the National Institutes of Health guidelines for the use of experimental animals and was reviewed and approved by the Animal Studies Ethics Committee of Yokohama City University. All experiments were performed according to the ARRIVE guidelines. Male C57BL/6 J wild-type mice were purchased from Oriental Yeast Co, Ltd. The mice were housed in a controlled environment with a 12:12 light/dark cycle and ambient temperature (25℃) and humidity. The mice were allowed free access to food and water. They were fed a standard diet (0.5% NaCl, 3.6 kcal/g, and 13.3% energy as fat; Oriental MF, Oriental Yeast Co, Ltd.).

### Experiment protocol and administration of aristolochic acid and etanercept

After 2 weeks of acclimatization, mice (9–11 weeks old) were randomly assigned to either the control group, AA group, or AA + ETN group (*n* = 8 mice per group) (Fig. 1a). The control group received an intraperitoneal (i.p.) injection of dimethyl sulfoxide (DMSO) and a subcutaneous (s.c.) injection of normal saline (NS). The AA group received an i.p. injection of AA (3 mg/kg) and an s.c. injection of NS^32,48^. The AA + ETN group received an i.p. injection of AA (3 mg/kg) and an s.c. injection of ETN (5 mg/kg)^49–51^. The ETN dose was determined from previous studies and our preliminary experiment (Supplementary Fig. S1 online). In the preliminary experiment, ETN at a lower dose (1 mg/kg, twice per week) did not exert any anti-fibrotic effect, thus we employed the higher dose (5 mg/kg, twice per week) for this study. Figure 1a shows the time course of the experiment. AA or DMSO was administered twice per week for 4 weeks. ETN or NS was administered twice per week for 8 weeks. Based on previous studies^32,48^, we adopted a protocol of a 4-week AA administration followed by a 4-week remodeling period, to evaluate kidney fibrosis in a more chronic phase.

### Blood pressure and heart rate measurements

Systolic BP and HR were measured via a noninvasive procedure using a computerized tail-cuff plethysmograph (MK-2000 BP monitor; Muromachi Kikai, Tokyo, Japan), as described previously^52^. All measurements were performed at the same time between 10:00 and 13:00 to avoid diurnal variation in BP. At least 10 BP and HR measurements were analyzed. Systolic BP and HR were measured 0, 4, and 8 weeks after the intervention (Fig. 1a).

### Metabolic cage analysis

A metabolic cage analysis was performed at the end of the experimental period, as described previously^53^. Mice were housed in metabolic cages for 2 consecutive days and given free access to food and water. A 24 h urine collection procedure was performed on day 2.

### Biochemical assays

Blood samples were collected by cardiac puncture when the mice were euthanized in the fed state. Whole-blood samples were centrifuged at 3,000 rpm at 4 °C for 10 min to separate plasma, which was snap-frozen and stored at -80 °C until use. Plasma Cr, BUN, and urinary Cr were measured using an autoanalyzer (Hitachi 7180; Hitachi, Tokyo, Japan). Urinary albumin was measured by an immunoturbidimetric assay (Fujifilm Wako Pure Chemical Corp., Tokyo, Japan).

### Histological and immunohistochemical analyses

Histological analyses were performed as described previously^54,55^. Briefly, mouse kidneys were fixed in 4% paraformaldehyde in PBS, incubated overnight at 4 °C, and embedded in paraffin. Sections (4 μm thick) were stained with periodic acid–Schiff (PAS) and PSR. The glomerular area was measured by tracing the outline of the glomerular tuft of at least 50 glomeruli in the cortical fields of PAS-stained specimens. Fibrotic areas were measured digitally using a fluorescence microscope (BZ‐X800; Keyence, Osaka, Japan) in the cortical fields of PSR-stained specimens. Immunohistochemistry was performed as described previously^56^. Sections were incubated with anti‐F4/80 antibodies (1:100; ab111101; Abcam, Cambridge, MA, USA). The TUNEL assay was conducted using an in situ apoptosis detection kit (MK-500; Takara Biomedicals, Tokyo, Japan). Interstitial TUNEL-positive cells were counted in 10 randomly selected cortical fields (magnification: × 200). All measurements were blinded.

### Real-time quantitative reverse transcription polymerase chain reaction (PCR) analysis

Total RNA was extracted from kidney tissues using ISOGEN (Nippon Gene, Tokyo, Japan), and cDNA was synthesized using the SuperScript III First-Strand System (Invitrogen, Carlsbad, CA, USA), according to the manufacturer’s protocol. Real-time quantitative reverse transcription PCR analysis was performed using an ABI PRISM 7000 Sequence Detection System by incubating the reverse transcription products with the TaqMan PCR Master Mix and designed TaqMan probes (Applied Biosystems, Foster City, CA, USA). The TaqMan probes used for PCR were Bax, Mm00432051_m1; Bcl-2, Mm00477631_m1; CD68, Mm03047343_m1; collagen type I (Col1a1), Mm00801666_g1; collagen type III (Col3a1), Mm01254476_m1; F4/80, Mm00802529_m1; IL-1β, Mm00434228_m1; IL-6, Mm00446190_m1; MCP-1, Mm00441242_m1; TGF-β, Mm01178820_m1; and TNF-α, Mm00443258_m1. The mRNA levels were normalized to the 18S rRNA control.

### Immunoblot analysis

Western blotting analysis was performed as described previously^56^. Briefly, kidney tissues were mechanically homogenized in SDS‐containing sample buffer with the complete protease inhibitor cocktail (Roche, Basel, Switzerland), and were denatured by heating at 95 °C for 5 min. Proteins were quantified using the RC DC protein assay kit (Bio-Rad, Hercules, CA, USA). Equal amounts of protein extract were separated by 5–20% sodium dodecyl sulfate–polyacrylamide gel electrophoresis (SDS-PAGE) and transferred to a polyvinylidene difluoride membrane using the iBlot Dry Blotting System (Invitrogen, Paisley, UK). The membranes were blocked with 5% skim milk for 1 h at room temperature and probed with specific primary antibodies to p38 MAPK (1:1000, sc-728 Santa Cruz Biotechnology, Santa Cruz, CA, USA), phosphorylated p38 MAPK (1:2000, V1211 Promega Corp., Madison, WI, USA). Horseradish peroxidase-conjugated goat anti-rabbit IgG or goat anti-mouse IgG secondary antibodies were added for 1 h at room temperature. The Immobilon Forte Western HRP substrate (Merck, Kenilworth, NJ, USA) was used for detection. The images were captured with auto-exposure, and automatically optimized using ChemiDoc Touch (Bio-Rad Laboratories).

### Calculations and statistical analysis

Statistical analyses were performed using Prism software version 9.0.1 (GraphPad Software, San Diego, CA, USA). The results are presented as means ± SEM. Differences in multiple comparisons were assessed by two-way repeated-measures analysis of variance (ANOVA) for repeated measures data over time, including BW, BP, and HR, and one-way ANOVA was used for the other data. Post hoc analysis was performed using a Dunnett’s test and Tukey’s test when two-way repeated-measures ANOVA or one-way ANOVA indicated a significant difference, respectively. *P* < 0.05 was considered significant.

## Supplementary Information

Supplementary Information.

## Data Availability

All relevant data are within the paper. The datasets are available from the corresponding authors upon reasonable request.
