# Supplementary material for: Effects of tumor necrosis factor-α inhibition on kidney fibrosis and inflammation in a mouse model of aristolochic acid nephropathy
Source: Sci Rep. 2021 Dec 8;11:23587. doi: 10.1038/s41598-021-02864-1 (PMC8654826; doi:10.1038/s41598-021-02864-1)
Supplement: Supplementary file 1 — Supplementary Information. [file 41598_2021_2864_MOESM1_ESM.docx]

**Effects of tumor necrosis factor-α inhibition on kidney fibrosis and inflammation in a mouse model of aristolochic acid nephropathy**

Shinya Taguchi^1^, Kengo Azushima^1*^, Takahiro Yamaji^1,2^, Shingo Urate^1^, Toru Suzuki^1^, Eriko Abe^1^, Shohei Tanaka^1^, Shunichiro Tsukamoto^1^, Daisuke Kamimura^1^, Sho Kinguchi^1^, Akio Yamashita^3^, Hiromichi Wakui^1*^ and Kouichi Tamura^1^

Shinya Taguchi and Kengo Azushima contributed equally to this work.

^1^ Department of Medical Science and Cardiorenal Medicine, Yokohama City University Graduate School of Medicine, Yokohama, Japan

^2^ Cardiovascular and Metabolic Disorders Program, Duke-NUS Medical School, Singapore

^3^ Department of Investigative Medicine, Graduate School of Medicine, University of the Ryukyus, Okinawa, Japan

Corresponding authors: Kengo Azushima, M.D., Ph.D. and Hiromichi Wakui, M.D., Ph.D.

Department of Medical Science and Cardiorenal Medicine

Yokohama City University Graduate School of Medicine

3-9 Fukuura, Kanazawa-ku, Yokohama, Japan 236-0004.

Tel.: 81-45-787-2635; Fax: 81-45-701-3738

E-mail: [azushima@yokohama-cu.ac.jp](mailto:azushima@yokohama-cu.ac.jp) or [hiro1234@yokohama-cu.ac.jp](mailto:hiro1234@yokohama-cu.ac.jp)


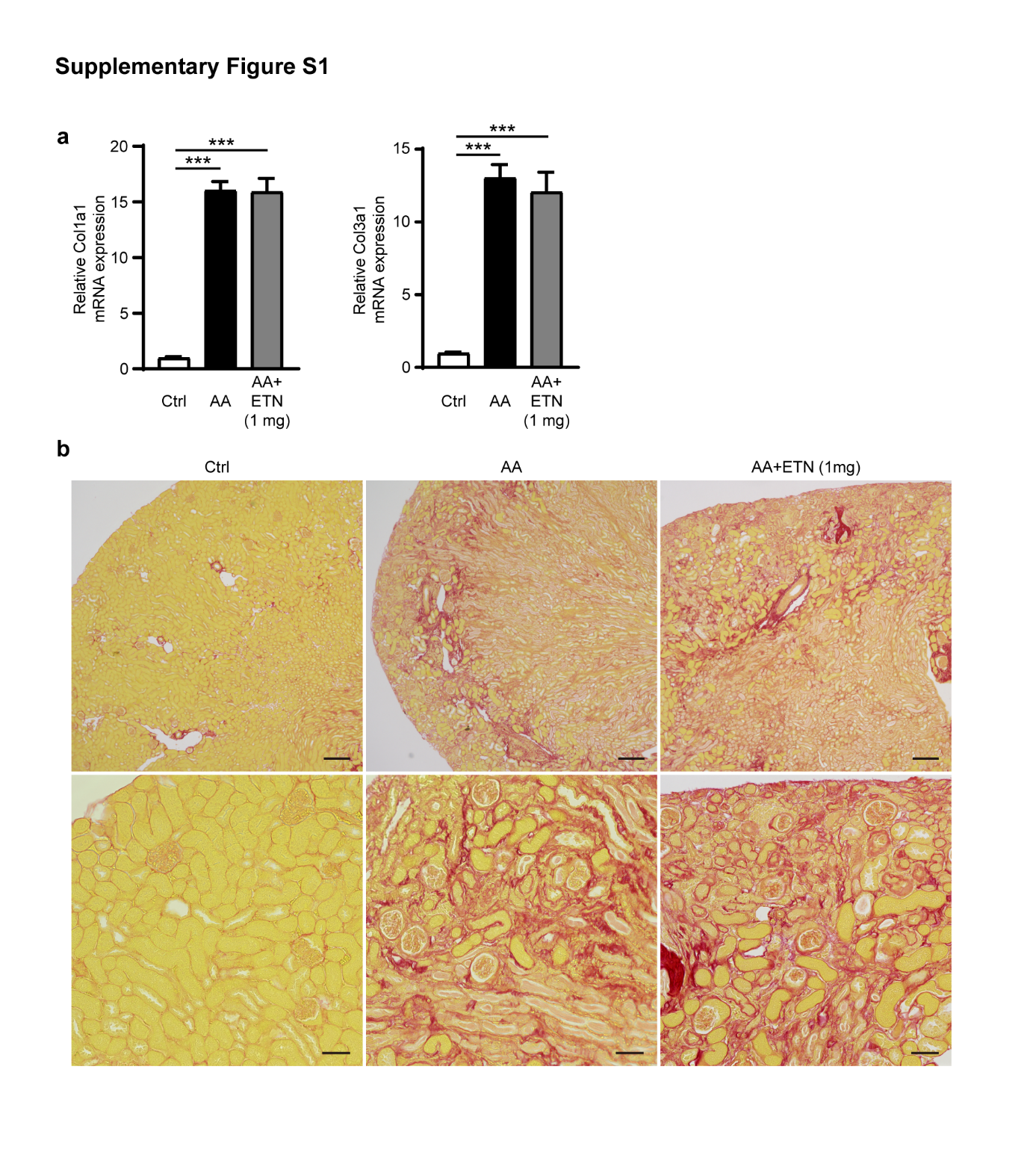


**Supplementary Figure S1.** Preliminary experiments investigating the effect of etanercept at a lower dose (1 mg/kg, twice per week) on kidney fibrosis. **(a)** Relative renal mRNA expression of Col1a1 and Col3a1 (n = 7–8). **(b)** Representative images of kidneys stained with picrosirius red (upper panel, original magnification, ×40, bar, 200 μm; lower panel, original magnification, ×200, bar, 50 μm). Values are means ± SEM. *** *P* < 0.001. Ctrl, control; AA, aristolochic acid; ETN, etanercept; Col, collagen.


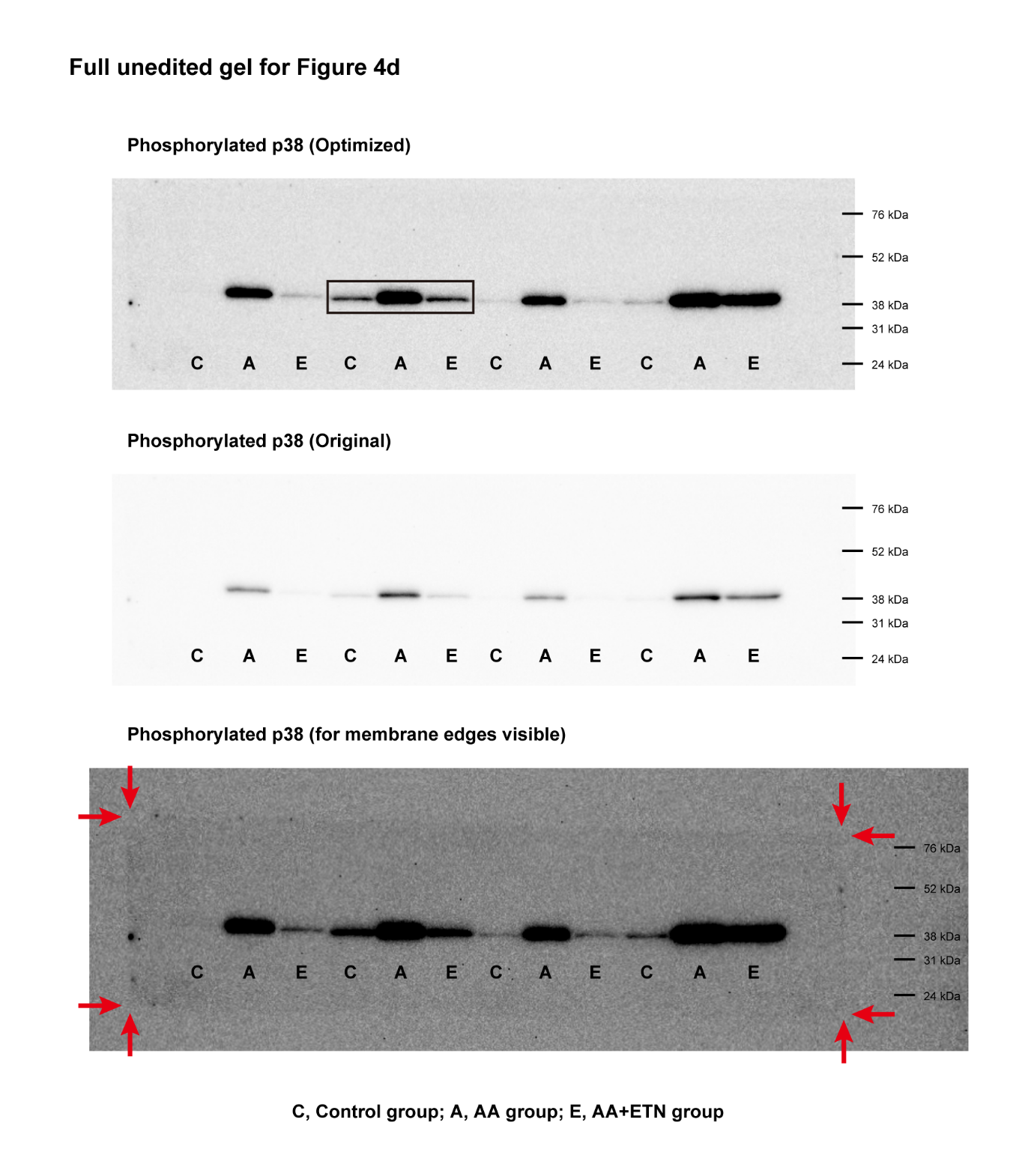


Full scan of the entire original blots incubated with phosphorylated p38 MAPK. Top panel is an image automatically optimized by ChemiDoc Touch (Bio-Rad Laboratories). Black box indicates the representative image shown in Fig. 4d. Middle panel is an original image with Gamma scale = 1.0. Since an image of the full-length membrane is absent, we provide bottom panel which is an image manually adjusted so that the membrane edges are visible. The samples were transferred to membranes in this sequence; Control group (C), AA group (A), and AA+ETN group (E).


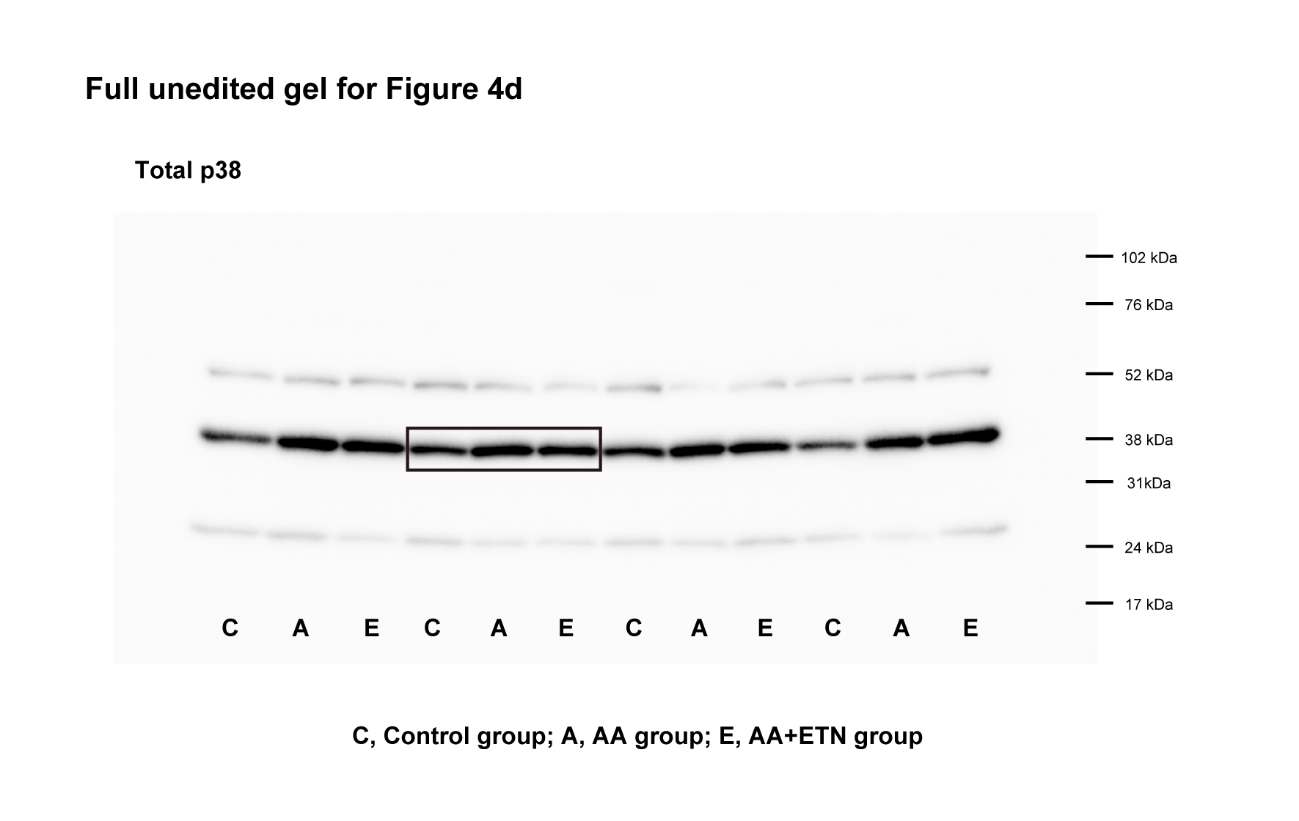


Full scan of the entire original blots incubated with total p38 MAPK. The samples were transferred to membranes in this sequence; Control group (C), AA group (A), and AA+ETN group (E).
